# Regulation of type VI secretion systems at the transcriptional, posttranscriptional and posttranslational level

**DOI:** 10.1099/mic.0.001376

**Published:** 2023-08-08

**Authors:** Julia Takuno Hespanhol, Luize Nóbrega-Silva, Ethel Bayer-Santos

**Affiliations:** ^1^​ Department of Microbiology, Institute of Biomedical Sciences, University of Sao Paulo, Sao Paulo 05508-900, Brazil

**Keywords:** bacterial antagonism, gene regulation, posttranscriptional regulation, posttranslational regulation, T6SS, transcription factors

## Abstract

Bacteria live in complex polymicrobial communities and are constantly competing for resources. The type VI secretion system (T6SS) is a widespread antagonistic mechanism used by Gram-negative bacteria to gain an advantage over competitors. T6SSs translocate toxic effector proteins inside target prokaryotic cells in a contact-dependent manner. In addition, some T6SS effectors can be secreted extracellularly and contribute to the scavenging scarce metal ions. Bacteria deploy their T6SSs in different situations, categorizing these systems into offensive, defensive and exploitative. The great variety of bacterial species and environments occupied by such species reflect the complexity of regulatory signals and networks that control the expression and activation of the T6SSs. Such regulation is tightly controlled at the transcriptional, posttranscriptional and posttranslational level by abiotic (e.g. pH, iron) or biotic (e.g. quorum-sensing) cues. In this review, we provide an update on the current knowledge about the regulatory networks that modulate the expression and activity of T6SSs across several species, focusing on systems used for interbacterial competition.

## Introduction

Bacteria live in complex polymicrobial communities and are constantly competing for resources. Diverse antagonistic mechanisms are used by bacteria to gain an advantage over other species, comprising systems that require or not direct contact between cells [1]. The type VI secretion system (T6SS) is a contact-dependent system that translocates toxic effector proteins inside target prokaryotic or eukaryotic cells [2]. T6SSs are encoded in the genome of around 25 % of all Gram-negatives, including plants and animal pathogens, symbionts or commensals, and environmental strains found in marine or terrestrial habitats [3, 4]. The great variety of bacterial species and environments occupied by such bacteria reflects the complexity of regulatory signals and networks that control the expression and activation of their T6SSs. At these diverse environments, bacteria can also alternate between free-living (planktonic) and surface-associated (biofilm) states, imposing another layer of complexity to the control of gene expression [5].

**Fig. 1.**
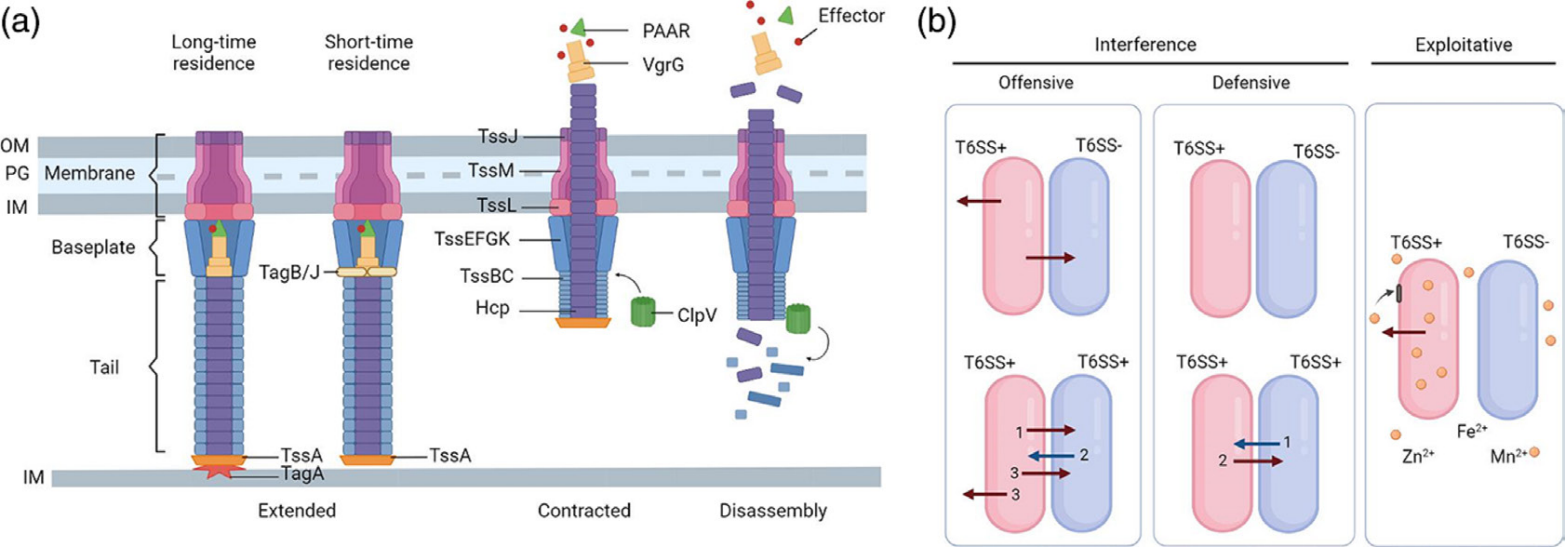
Structure and mode of action of different T6SSs. (**a**) Schematic representation of T6SS apparatus. T6SS is divided into three complexes: membrane, baseplate and tail. The membrane complex is formed by TssJML; the baseplate is formed by TssEFGK; the tail is composed by a sheath of TssBC with an internal tube of hexamers of Hcp topped with a trimer of VgrG and a PAAR protein. Assembly of the tail is facilitated by TssA. The system is stabilized at the distal end by TagA in long-time residence systems or is stabilized at the baseplate by TagB or TagJ in short-time residence systems. Contraction of the sheath propels the inner tube, delivering effectors into target cells or the extracellular medium. After contraction, the ATPase ClpV is responsible for depolymerizing the sheath. IM: inner membrane; OM: outer membrane; PG: peptidoglycan. (**b**) Interference and exploitative T6SS systems. Interference systems are divided into offensive and defensive. Offensive T6SSs fire randomly at neighbour cells without a prior attack (red arrows). Some offensive systems can be regulated to increase firing after an attack (blue arrow). Numbers represent the order of firing events. Defensive T6SSs require an initial attack (blue arrow) for activation. Exploitative T6SSs secrete effectors in the medium to chelate metal ions (yellow spheres), which will be later internalized by specialized transport systems. Created with BioRender.com.

The T6SS is a contractile nanomachine structurally and mechanistically related to the tail of T4 bacteriophages [6]. This system is composed of several structural proteins that assemble into three major complexes: membrane, baseplate and tail (Fig. 1a) [7]. The membrane complex is formed by the proteins TssJ (type VI secretion system subunit J), TssL and TssM. The baseplate is composed of TssE, TssF, TssG and TssK [8, 9]. An uncharacterized signal leads to a conformational change at the baseplate and contraction of the sheath formed by TssB and TssC [10, 11], which ejects an inner tube composed of Hcp hexamers towards target cells [12, 13]. At the tip of this inner tube there is a trimer of VgrG (valine-glycine repeat protein G), which is covered by a protein with a PAAR domain (proline-alanine-alanine-arginine) [14, 15]. TssA is a cap protein that participates in the assembly of the tail [16], and ClpV (caseinolytic peptidase V) is an ATPase that disassembles the system after each contraction event [17].

The T6SS apparatus itself is not known to possess cytotoxic properties, and it is the biochemical properties of secreted effector toxins delivered into target cells via this apparatus that are responsible for its function. These effectors can be secreted (1) as an extended C-terminal domain of the structural components Hcp, VgrG and PAAR, which classify them as evolved or specialized effectors; or (2) associated with these structural components with or without the help of an adaptor protein, which classify them as cargo effectors [18, 19]. These effectors act on important structural components and cellular processes in the prey cells [20]. Bacteria that encode antibacterial toxins also produce a cognate immunity protein in the same operon to protect them against self-intoxication and prevent killing of sister cells [21]. In addition, T6SS effectors can target eukaryotic cells [22, 23], or be secreted extracellularly to contribute to the scavenging of scarce metal ions [24]. Most T6SS structural proteins are encoded in a single main cluster, while genes encoding proteins from the inner tube (Hcp, VgrG and PAAR), effectors and their cognate immunities are also encoded in different genomic loci, comprising orphan or auxiliary clusters [25, 26].

The T6SSs can contribute to bacterial competition and fitness in two distinct ways: (1) via interference competition in which the T6SS secrete antibacterial toxins to kill or inhibit the growth of competitors; or (2) via exploitative competition in which the system secrete effectors that promote the uptake of scarce metal ions (iron, zinc, copper, manganese and molybdate) via another membrane transport system (Fig. 1b) [27–32]. The T6SSs involved in interference can be further classified into offensive and defensive systems according to the regulatory mechanisms that control their activation (Fig. 1b). Offensive T6SS apparatuses assemble, contract and reassemble constantly and in different locations within the cell, firing toxic effectors against neighbour cells without any prior aggression from the surrounding [33]. Conversely, defensive T6SSs fire toxic effectors only after a previous attack that signals to trigger a counterattack [33, 34]. Such an activation seems to be related to membrane damage [35] and is induced by the mechanical activity of another T6SS [34], the action of effectors with phospholipase activity [36], the conjugative type 4 secretion system (T4SS) [37], and chemical substances such as antibiotics [37], DNA and chelators [38]. In addition, exploitative systems enhance bacterial fitness via increased acquisition of essential nutrients and are mainly regulated by the concentration of ions in the medium [30, 31]. It is worth noting that some T6SSs display dual function, working as antibacterial and exploitative systems [39] or antibacterial and antieukaryotic systems [23, 26, 40], and some bacteria encode more than one T6SS in their genomes [29, 41].

Many T6SS gene clusters are encoded within pathogenicity islands, which were probably acquired by horizontal gene transfer events [4] and integrated into preexisting regulatory networks providing an advantage to the bacteria. Over the years since its discovery, studies have revealed that the regulation of T6SSs occurs at three different levels, depending on the bacterial species: transcriptional, posttranscriptional and posttranslational [42, 43]. Transcriptional regulation involves factors such as quorum-sensing (QS) systems, two-component systems (TCS), alternative sigma factors and histone-like proteins. Posttranscriptional regulation engages RNA-binding proteins and small regulatory RNAs that control the stability and translation of mRNAs. Finally, posttranslational regulation relies mainly on phosphorylation of T6SS structural proteins to regulate system assembly. In this review, we provide an update on the current knowledge regarding the environmental signals, regulatory proteins and networks that modulate the expression and activity of antibacterial and exploitative T6SSs across several species. As this review was designed for a special collection focusing on systems used for interbacterial competition, T6SSs that display only anti-eukaryotic activity were not included.

## Environmental signals controlling the expression of T6SS

Several abiotic and biotic signals inform bacteria about the environmental conditions to determine which genes must be expressed at a given time. T6SSs are regulated by abiotic factors such as temperature, osmolarity, pH, oxygen and the concentration of metal ions. In addition, biotic signals like host-derived chitin and mucin, quorum sensing molecules, antibiotics and toxic effectors also modulate T6SS expression.

Temperature and osmolarity are key factors controlling the expression of T6SSs in different organisms. At low osmolarity, the T6SS cluster of *Vibrio cholerae* O1 is repressed, but under high osmolarity its expression is upregulated and secretion of Hcp is detected in the medium [44]. *V. cholerae* O1 does not display antibacterial activity at 15 °C but killing is observed in temperatures ranging between 25 and 37 °C [45]. Such osmolarity and temperatures are similar to the conditions found in statuary environments where *V. cholerae* can be found [46]. In *Vibrio parahaemolyticus,* the antibacterial T6SS1 is induced by warm marine-like conditions or upon surface sensing [47, 48]. Meanwhile, the K-1 T6SS of *Pseudomonas putida* is repressed after surface attachment [49]. In *Vibrio fischeri *, the T6SS2 is upregulated in environments with high viscosity and neutral to acidic pH, which mimics the conditions in the squid host [50]. In addition, *Klebsiella pneumoniae* upregulates its antibacterial T6SS (locus 1) upon changes in conditions of the medium, such as increase in salt concentrations and reduction of pH [51]. The phytopathogen *Agrobacterium tumefaciens* upregulates its T6SS and promotes bacterial killing in acidic media (pH 5.5), which is one of the conditions found at plant wound sites [52].

**Fig. 2.**
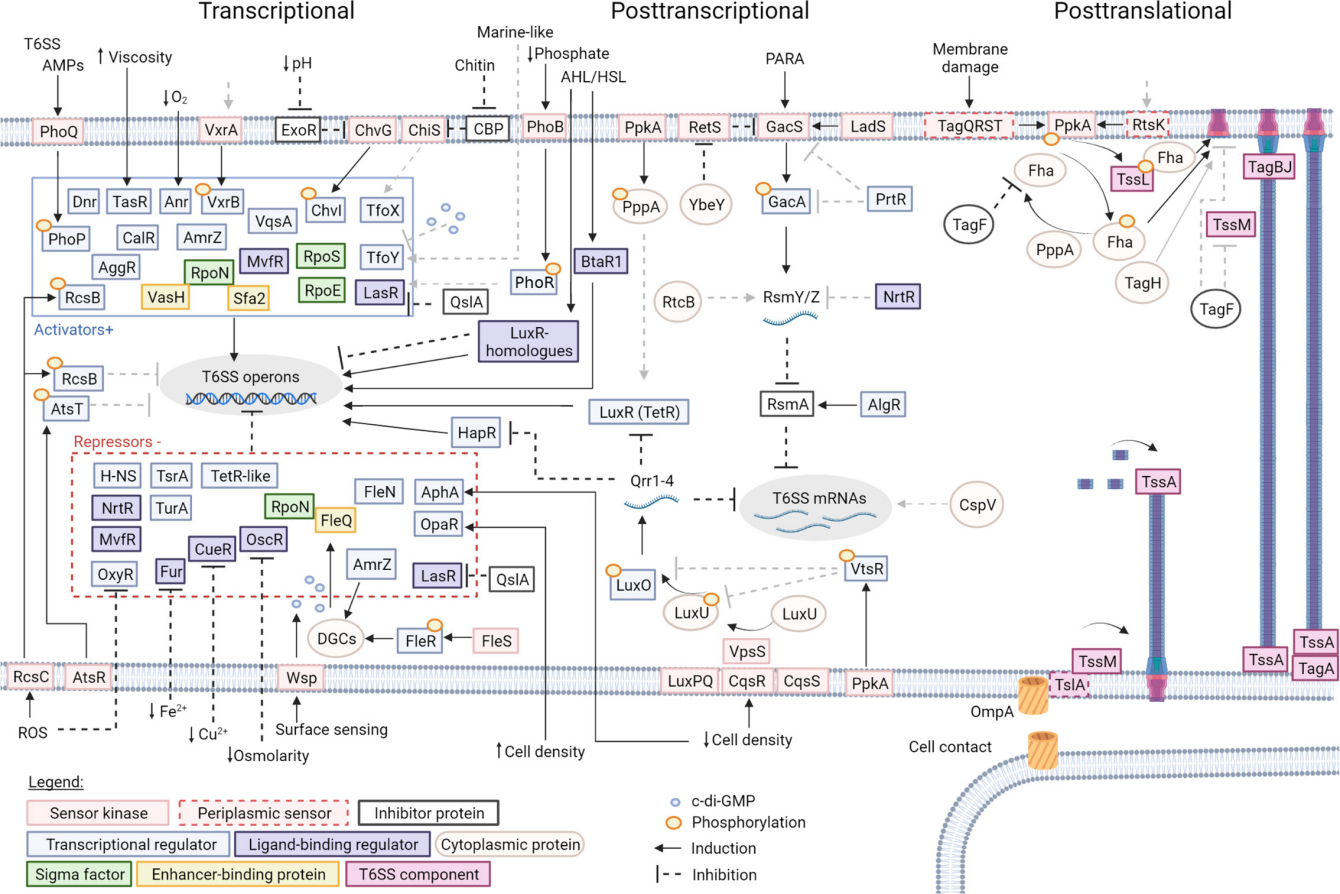
The regulatory pathways controlling expression of T6SSs. Schematic representation of regulatory pathways described in several bacterial species depicted at three levels: transcriptional, posttranscriptional and posttranslational. Several environmental conditions and events trigger the initial components of many cascades: AMPs (antimicrobial peptides); AHL/HSL (N-acylhomoserine lactone and homoserine lactone); PARA (* Pseudomonas aeruginosa* response to antagonism); ROS (reactive oxygen species). Black continuous lines with arrows represent known induction/activation, while black dashed lines represent known inhibition/repression. Grey dashed lines represent possible/unknown induction or inhibition. Pairs of sensor kinases and associated transcriptional regulators are the following: PhoQ/PhoP (phosphatase metabolism gene Q/P); PhoB/PhoR (phosphatase metabolism gene B/R); VxrA/B (* Vibrio* type six regulator A/B); ChvG/ChvI (chromosomal virulence gene G/I); FleS/FleR (flagellar expression sensor/regulator); Wsp system (wrinkly spreader phenotype); RcsC/RcsB (regulation of capsular polysaccharide synthesis C/B); AtsR/AtsT (adherence and T6SS regulator); ChiS (chitin degradation sensor); TfoX (transformation gene); RetS (regulator of exopolysaccharides and type III secretion); GacS/GacA (global activator gene S/A); LadS (lost adherence gene S); TagQRST (type VI accessory genes QRST); PpkA (putative protein kinase A); RtkS (regulator of T6SS kinase in *Serratia *); LuxPQ (luminescence related gene P/Q); CqsR (cholerae quorum-sensing receptor); CqsS (cholerae quorum-sensing sensor); VpsS (* Vibrio* polysaccharide biosynthesis sensor). Additional transcriptional regulators are as follows: TasR (type VI associated transcriptional regulator); Anr (anaerobic regulator of arginine deiminase and nitrate reductase); Dnr (dissimilative nitrate respiration regulator); VqsA (* Vibrio* quorum-sensing activator); TfoY (transformation gene X homologue); AmrZ (alginate and motility regulator); AggR (enteroaggregative *E. coli* regulator); H-NS (histone-like nucleoid structuring proteins); TurA (TOL upper operon repressor A); TsrA (type VI secretion system regulator A); OxyR (hydrogen peroxide sensor and transcription activator); TetR-like (tetracycline regulator); AphA (activator of *tcpP* and *tcpH* expression); OpaA (opacity regulator); LuxO (transcriptional regulator); HapR (HA/protease gene regulator); AlgR (alginate biosynthesis regulator); VtsR (* Vibrio* type six secretion regulator). Ligand-binding regulators are as follows: MvfR (multiple virulence factor regulator); LasR (regulator of *lasB*); NrtR (Nudix-related transcriptional regulator); Fur (ferric uptake regulator); CueR (copper export regulator); FleN (flagellar expression gene N); LuxR-homologues (luminescence regulator); BtaR1 (*Burkholderia thailandensis luxR* homologue). Sigma factors and EBP: RpoN (σ^54^); RpoS (σ^38^); RpoE (σ^24^); VasH (virulence-associated secretion gene H); FleQ (flagellar expression gene Q); Sfa2 (sigma factor activator 2). Cytoplasmic proteins are as follows: PppA (putative protein phosphatase A); YbeY (RNase); RtcB (RNA terminal phosphate cyclase gene B); CspV (cold shock protein V); Fha (Fork-head associated protein): TagH (type VI accessory gene H); DGCs (diguanylate cyclases). Inhibitor proteins are as follows: ExoR (exopolysaccharide synthesis repressor); CBP (chitin binding protein); QslA (QS LasR-specific anti-activator); RsmA (repressor of secondary metabolites gene A); TagF (type VI accessory gene F). T6SS components: TslA (type six secretion dynamic localization protein A); TssM (type VI secretion system subunit M); TssA (type VI secretion system subunit A); TagA (type VI accessory gene A); TssL (type VI secretion system subunit L); TagB/J (type VI accessory genes B/J). Regulatory small RNAs are as follows: RsmY/N (repressor of secondary metabolites gene Y/N); Qrr1-4 (quorum-sensing regulatory sRNA 1–4). Created with BioRender.com.

The concentration of oxygen and reactive oxygen species (ROS) are important environmental signals in the control of T6SSs. Oxygen can directly interact and control the activity of transcriptional regulators, such as Anr (anaerobic regulator of arginine deiminase and nitrate reductase)*,* which belongs to the Fnr-family (fumarate and nitrate reductase regulator) of transcriptional regulators [53]. In the absence of oxygen, Anr dimerizes and binds to promoter regions to control transcription [54]. Under anaerobic conditions, Anr induces the expression of the antibacterial and exploitative H2-T6SS from *Pseudomonas aeruginosa* [55]. Anr also promotes the expression of the transcriptional regulator Dnr (dissimilative nitrate respiration regulator) from the Fnr-family, which further promotes H2-T6SS expression [56]. This system secretes the effector ModA, a molybdate chelator that helps import ion used in the nitrate respiratory pathway under anaerobic conditions [55]. OxyR (hydrogen peroxide sensor and transcription activator) is a response regulator that in the presence of H_2_O_2_ is oxidized and binds to promoter regions to induce the activation of gene expression [57], and it was shown to upregulate the exploitative T6SS-4 of *Burkholderia thailandensis* (Fig. 2) [29]. In the presence of ROS, T6SS-4 is upregulated and secretes the effector TseM (T6SS-secreted Mn^2+^-binding protein), which binds to extracellular Mn^2+^ and is transported back to the cell via the TonB-dependent transporter MnoT (Mn^2+^-specific outer membrane transporter) [29].

The concentration of ions in the medium is an additional factor controlling the expression of T6SSs. Some transcriptional regulators respond to external ion concentrations like the global regulator Fur (ferric uptake regulator), which is involved in iron homeostasis [58]. In the presence of iron, Fur dimerizes and binds to a specific sequence in the promoter regions (Fur boxes) to control gene transcription [58]. In *Salmonella* Typhimurium, Fur was shown to repress the T6SS by directly binding to *clpV* promoter region [59]. Fur has also been shown to repress the T6SS of *Edwardsiella tarda* [60], enteroaggregative *E. coli* [61, 62], avian pathogenic *E. coli* [63], *P. aeruginosa* [64] and *Cupriavidus necator* [31]. Conversely, Fur was reported to be required for the upregulation of the T6SS of *K. pneumoniae,* as the Δ*fur* mutant did not kill competing bacteria in a T6SS-dependent manner (Fig. 2) [51]. Another transcriptional regulator displaying a similar mechanism is CueR (copper export regulator), which represses the antibacterial and exploitative H2-T6SS of *P. aeruginosa* by direct binding to the promoter regions in the presence of copper (Fig. 2) [30]. However, under low Cu^2+^ concentration, H2-T6SS is upregulated and secretes the Cu^2+^-binding effector Azu extracellularly, which will chelate Cu^2+^ and be internalized via OprC (outer membrane porin copper transport) [30]. In addition to metal ions, low concentrations of phosphate are also responsible for inducing the expression of the H2- and H3-T6SS of *P. aeruginosa* [65]. In *V. fischeri *, the combination of neutral pH and Ca^2+^ at seawater-like or host-like concentration is able to induce T6SS2-mediated bacterial killing [66].

In intestinal pathogens such as *S*. Typhimurium, *V. cholerae* and *Shigella sonnei *, the T6SS is important for host colonization via competition against the resident microbiota [67–69]. The T6SSs of these bugs is regulated by host-produced signals such as bile salts and mucins, which positively regulate the T6SS activity of *S*. Typhimurium and *V. cholerae* [68, 70]. In a similar manner, *V. cholerae* is commonly associated with aquatic animals and its T6SS gene cluster is upregulated by chitin [71].

In addition to host-derived signals, prokaryotic made cues are key players in regulating T6SSs. QS is a form of bacterial communication guided by cell density that allows the synchronized regulation of a variety of behaviours via tightly controlled gene expression [72]. QS is known to regulate T6SS gene expression in several organisms such as *Vibrio* spp. [73–75]*, Aeromonas hydrophila* [76], *Chromobacterium violaceum* [77], *Enterobacter cloacae* [78] and *P. aeruginosa* [79]. Besides friendly QS molecules, hostile biotic signals such as the production of antibiotics and T6SS effectors are important clues coordinating the regulation of T6SSs. It has been shown that polymyxin B, an acylated decapeptide antibiotic causing membrane damage, induces expression of the T6SS in *E. coli* [80]. In *P. aeruginosa *, the aminoglycoside antibiotic kanamycin also activates the T6SS [81]. The diverse responses to different antibiotics indicate that these molecules can function as signals triggering bacteria to assemble defence mechanisms [82]. As mentioned above, defensive T6SSs are activated by the mechanical activity of another T6SS [34] and the action of effectors with phospholipase activity [36]. All these signals start a downstream cascade that is regulated at different levels.

## Transcriptional regulation

Regulation of gene expression at the transcriptional level is the first layer controlling the activation of T6SSs. Transcription of T6SS gene clusters is induced by several pathways that include varied response regulators (Fig. 2) (Table 1). These pathways can involve (1) TCS composed of a sensor histidine-kinase that phosphorylates a cytoplasmic response regulator to modulate gene transcription [83]; (2) phosphorelay systems in which a phosphorylation cascade starting with a membrane sensor leads to activation of a cytoplasmic transcriptional regulator [84]; (3) a variety of transcriptional regulators controlled by different pathways or direct binding to specific ligands; (4) sigma factors that are part of and control the activity of RNA polymerases [85]; and (5) histone-like nucleoid structuring proteins (Fig. 2) (Table 1) [86].

**Table 1.** List of bacterial species encoding T6SSs, the environmental cues and regulatory components controling system expression and activity

Species	T6SS cluster	Type	Conditions	Regulation	Regulatory system	Level	References
* Acinetobacter baumannii *	–	Antibacterial (Offensive)	–	–	H-NS (H-NS family); TetR-like (TetR family)	Transcriptional	[145–147]
			Assembly at sites with cell-cell contact	–	TslA; OmpA	Posttranslational	[211]
* Acinetobacter baylyi *	–	Antibacterial	Assembly at sites with cell-cell contact	–	TslA; OmpA	Posttranslational	[211]
* Aeromonas hydrophila *	–	Antibacterial	–	–	LuxR homolog (LuxR family); VasH	Transcriptional	[76, 152, 153]
* Agrobacterium tumefaciens *	–	Antibacterial	↓pH	Up↑	ExoR; ChvG/ChvI (OmpR family)	Transcriptional	[52, 95]
			–	–	TPP; TagF	Posttranscriptional	[205]
* Burkholderia cenocepacia *	T6SS-1	Antibacterial	–	–	AtsT/AtsR	Transcriptional	[98, 99]
			–	–	CepR (LuxR family); CciR (LuxR family)	Transcriptional	[112, 113]
* Burkholderia thailandensis *	T6SS-1	Antibacterial	↑Cell density	Up↑	BtaR1 (LuxR family)	Transcriptional	[41]
			Assembly at sites with cell-cell contact	–	TagM1 (TslA-like protein)	Posttranslational	[211]
* Burkholderia thailandensis *	T6SS-4	Exploitative	ROS	Up↑	OxyR (LysR family)	Transcriptional	[29]
* Chromobacterium violaceum *	–	Antibacterial (Offensive)	–	–	CviR (LuxR family)	Transcriptional	[77]
* Cupriavidus necator *	T6SS-1	Exploitative	↓Fe^2+^	Up↑	Fur (Fur family)	Transcriptional	[31]
* Edwardsiella tarda *	–	Antibacterial Anti-Eukaryotic	↓Fe^2+^	Up↑	Fur (Fur family)	Transcriptional	[23, 60]
* Enterobacter cloacae *	T6SS-1	Antibacterial	↑Cell density	Up↑	SidA (LuxR family)	Transcriptional	[78]
* Escherichia coli* (EAEC)	T6SS-1	Antibacterial	↓Fe^2+^	Up↑	Fur (Fur family)	Transcriptional	[61, 62]
* Escherichia coli* (EAEC)	T6SS-3	Antibacterial	–	–	AggR (AraC family)	Transcriptional	[23, 143]
			T6SS attack	Up↑	–	Posttranslational	[144]
			–	–	TagA	Posttranslational	[212]
* Escherichia coli* (APEC)	T6SS-1	Antibacterial	↓Fe^2+^	Up↑	Fur (Fur family)	Transcriptional	[63]
* Escherichia coli* ExPEC	T6SS-1	Antibacterial Anti-Eukaryotic	–	Up↑	Rcs system	Transcriptional	[80]
* Klebsiella pneumoniae *	T6SS (locus I)	Antibacterial	↑NaCl ; ↓pH; attack from neighbours	Up↑	Fur (Fur family); RpoS (σ^70^); RpoN (σ^54^); PhoQ/PhoP (OmpR family); Hfq; PmrB/PmrA; H-NS	Trasncriptional	[51]
* Plesiomonas shigelloides *	–	Antibacterial	–	–	RpoN (σ^54^)	Transcriptional	[156]
* Pseudomonas aeruginosa *	H1-T6SS	Antibacterial (Defensive)	–	–	RpoN (σ^54^); FleS/FleR (NtrC family); LasR (LuxR family)	Transcriptional	[79, 91, 160]
			Membrane damage	Up↑	TPP pathway; TagF; TagJ	Posttranslational	[33, 35, 36, 38, 210]
			↑Sister cells lysis	Up↑	Gac/Rsm; RetS; RsmY, RsmZ; RsmA; PrtR; YbeY; NrtR (NrtR family); AlgR	Posttranscriptional	[88, 175, 178, 180, 181, 183, 184, 187]
* Pseudomonas aeruginosa *	H2-T6SS	Antibacterial Exploitative Anti-Eukaryotic	Stationary growth phase	Up↑	MvaT (H-NS family); AmrZ (Arc family); QslA; RpoN (σ^54^); Sfa2; LasR (LuxR family)	Transcriptional	[23, 64, 88, 159, 160, 167]
			↓Phosphate	Up↑	PhoR/PhoB	Transcriptional	[65]
			–	–	RsmA	Posttranscriptional	[88]
			↓Cu^2+^	Up↑	CueR (MerR family); RpoN (σ^54^)	Transcriptional	[30, 160]
			↓O_2_	Up↑	Anr (Fnr family); Dnr (Fnr family)	Transcriptional	[53, 55, 56]
* Pseudomonas aeruginosa *	H3-T6SS	Antibacterial Exploitative Anti-Eukaryotic	–	–	RpoN (σ^54^); Fur (Fur family); MvaT (H-NS family); LasR (LuxR family)	Transcriptional	[23, 28, 64, 160, 167]
			↓Phosphate	Up↑	PhoR/PhoB	Transcriptional	[65]
			–	–	RsmA	Posttranscriptional	[88]
			–	–	–	–	[28]
* Pseudomonas fluorescens *	F1-T6SS	Antibacterial	–	–	FleQ; RpoN (σ^54^)	Postranslational	[155]
* Pseudomonas fluorescens *	F3-T6SS	Antibacterial	–	–	FleQ; RpoN (σ^54^)	Transcriptional	[155]
* Pseudomonas plecoglossicida *	T6SS-2	Antibacterial	–	–	RpoE (σ^24^)	Transcriptional	[162]
* Pseudomonas putida* KT2440/KT2442	K1-T6SS	Antibacterial (Offensive)	Stationary growth phase	Up↑	–	Transcriptional	[89]
			–	–	FleQ; RpoN (σ^54^); RpoS (σ^38^); Wsp	Transcriptional	[49, 89, 157]
			–	–	TagB	Postranslational	[214]
			–	–	GacS/GacA; RetS	Postranslational	[89]
* Pseudomonas putida* KT2440/KT2442	K2-T6SS	Antibacterial	–	–	TurA (H-NS family)	Transcriptional	[168]
* Pseudomonas syringae *	H2-T6SS	Antibacterial	–	–	Sfa2; Rets; LadS; Gac/Rsm	Transcriptional	[90, 221]
* Salmonella* Typhimurium	SPI-6	Antibacterial	↓Fe^2+^	Up↑	Fur (Fur family)	Transcriptional	[59]
			–	–	H-NS (H-NS family)	Trancriptional	[166]
* Serratia marcescens* RM66262	–	Antibacterial (Offensive)	T6SS attack	Up↑	Rcs system	Transcriptional	[109]
* Serratia marcescens* Db10	–	Antibacterial (Offensive) Anti-eukariotic	Constitutively active	–	TPP pathway	Posttranslational	[23, 110, 204]
* Vibrio alginolyticus *	T6SS1	Antibacterial	–	Up↑	VqsA (LysR family)	Transcriptional	[75]
* Vibrio alginolyticus *	T6SS2	Antibacterial	–	Up↑	PpkA; LuxR (TetR-like); VtsR	Posttranslational	[125, 126]
* Vibrio cholerae* O1	–	Antibacterial Anti-Eukaryotic	↓NaCl	Down↓	OscR (IclR family)	Transcriptional	[23, 44]
			↑Cell density	Up↑	TrsA (H-NS family); LuxO (NtrC family); Qrr (sRNA); HapR (TetR family)	Transcriptional	[74, 120, 121]
			↑NaCl; Temp. 23–37 °C	Up↑	RpoN (σ^54^); VxrA/VxrB (OmpR family)	Transcriptional	[44, 100]
			Chitin	Up↑	ChiS; HapR (TetR family); TfoX (TfoX family); QstR	Transcriptional	[71]
			–	–	TfoY (TfoX family)	Transcriptional	[46, 196]
			Temp. 25–37 °C	Up↑	CspV	Posttranscriptional	[45]
* Vibrio cholerae* non-O1/O139	–	Antibacterial (Offensive)	–	–	TagH	Posttranslational	[208]
* Vibrio cholerae* V52	–	Antibacterial (Offensive)	–	–	VxrA/VxrB (OmpR family); VasH (σ^54^); TfoY (TfoX family)	Transcriptional	[23, 100, 101, 196, 218]
			–	–	TagA	Posttranslational	[213]
* Vibrio fluvialis *	T6SS2	Antibacterial	Temp. 25–30 °C; late-log to early stationary	Up↑	VasH; CqsS; HapR (TetR family)	Transcriptional	[123, 128]
			↑Cell density	Up↑	VfqR (LuxR family)	Transcriptional	[124]
* Vibrio fischeri *	T6SS2	Antibacterial	–	–	VasH-RpoN (σ^54^)	Transcriptional	[154]
			↑Viscosity; ↓pH	Up↑	TasR (Lrp family)	Transcriptional	[50, 140]
* Vibrio parahaemolyticus *	T6SS1	Antibacterial	Marine-like and surface sensing	Up↑	VP1391; VP1407 (Lrp family); TfoY (TfoX family)	Transcriptional	[47, 48]
			Mid-log phase	Up↑	ToxR (OmpR family); OpaR (TetR family); AphA (PadR family)	Transcriptional	[73]
			–	–	H-NS (H-NS family)	Transcriptional	[222]
* Vibrio parahaemolyticus *	T6SS2	Antibacterial	–	Up↑	CalR (LysR family); TfoX (TfoX family)	Transcriptional	[48, 73]
			–	Up↑	RpoN (σ^54^); OpaR (TetR family)	Transcriptional	[48, 223]
* Yersinia pseudotuberculosis *	T6SS4	Exploitative	–	–	RpoS (σ^38^)	Transcriptional	[161]

TCSs are part of a complex regulatory network to activate the three T6SSs of *P. aeruginosa *. This bacterium encodes three T6SS named H1-, H2- and H3-T6SS, which participate in bacterial killing, virulence towards eukaryotic cells and ion acquisition [39]. The H1-T6SS is the most well-studied example of a defensive system and presents the ‘tit-for-tat’ response, a behaviour in which the organism responds with reciprocity to neighbours’ attacks [33, 87]. The H1-, H2- and H3- T6SS of *P *. aeruginosa, the K1- T6SS of *P. putida* and the T6SS of *P. fluorescens* are positively controlled by the TCS GacS/GacA (global activator) [88–90]. In *P. aeruginosa,* the TCS FleS/FleR (flagellar expression sensor/regulator) induces expression of the global regulator AmrZ (alginate and motility regulator) [91], which belongs to the Arc-family of regulators and encode a ribbon-helix-helix DNA-binding domain and an oligomerization domain important for activity [92]. AmrZ directly controls the expression of the three T6SSs in *P. aeruginosa* by binding to the promoter region of structural components [88]. The contribution of AmrZ to the control of gene expression is dependent on the bacterial strain: in *P. aeruginosa* PA14 the H1- and H3-T6SS are induced by AmrZ while the H2-T6SS is repressed [88]; conversely, in PAO1 the H1-T6SS is repressed by AmrZ [91]. This repression occurs indirectly via the expression of diguanylate cyclases (DGC) responsible for the synthesis of the second messenger c-di-GMP that interacts with the transcriptional regulator FleQ to repress the H1-T6SS (Fig. 2) [91]. Similarly, in *P. putida* the K1-T6SS is repressed by c-di-GMP-FleQ in complex with the ATPase FleN [49]. Such downregulation is caused by the sensor system Wsp (wrinkly spreader phenotype), which upon surface attachment induces the downstream phosphorylation of the diguanylate cyclase WspR [93], thus promoting c-di-GMP synthesis and T6SS repression [49]. At the same time, the complex FleQ-FleN bound to c-di-GMP induces biofilm formation; hence, c-di-GMP levels control the switch between planktonic state with an active T6SS and biofilms with low antibacterial activity [49, 94]. These are examples of the complex regulatory networks that control bacterial behaviours.

The TCS ChvG/ChvI of *A. tumefaciens* is composed by the sensor kinase ChvG and the response regulator ChvI. ChvG is inhibited by the periplasmic repressor ExoR (exopolysaccharide synthesis repressor) at neutral pH, but in acidic conditions ExoR is degraded, allowing the autophosphorylation of ChvG and subsequent activation of the response regulator ChvI to induce T6SS expression [52, 95]. In addition, *A. tumefaciens* T6SS is inhibited by higher concentrations of c-di-GMP that are produced after an unidentified environmental stimulus. Curiously, the conjugative T4SS is also repressed by high levels of c-di-GMP [96]. The T6SS (locus I) of *K. pneumoniae* is regulated by the TCS PhoP/PhoQ in which the sensor kinase PhoQ detects antimicrobial molecules (e.g. ROS and antibiotics), leading to phosphorylation of the transcriptional regulator PhoP and T6SS activation [51, 97]. In *Burkholderia cenocepacia,* the sensor kinase AtsR (adherence and T6SS regulator) from the TCS AtsR/AtsT was shown to repress its antibacterial T6SS-1 [98, 99]; however, the external signal promoting kinase activation was not determined (Fig. 2). In the offensive T6SS of *V. cholerae* V52*,* the TCS VxrA/VxrB (* Vibrio* type six regulator) upregulates biofilm formation and T6SS expression [100, 101]; although the specific signal that activates VxrA was not yet identified (Fig. 2) [101].

Chitin recognition and signalling in *V. cholerae* O1 is mediated by a TCS composed of a histidine kinase ChiS (chitin degradation sensor) that is repressed by a chitin-binding protein (CBP) [102]. In the presence of chitin polymers, ChiS is derepressed and activates an unknown cytoplasmic regulator that leads to the expression of the transcriptional regulator TfoX (transformation gene) [103, 104], which induces T6SS gene expression [71]. T6SS expression in *V. cholerae* O1 is also dependent on the presence of the regulator HapR (HA/protease gene regulator) and the quorum-sensing intermediate regulator QstR (QS TfoX-dependent regulator) (Fig. 2) (Table 1) [71, 105]. In addition to positively regulating the T6SS via TfoX, the presence of chitin in the environment upregulates the genes controlling natural competence in *V. cholerae *, thus synchronizing target cell killing and DNA uptake to promote horizontal gene transfer [71, 106].

Members of the Enterobacteriaceae have an exclusive phosphorelay pathway called Rcs (regulation of capsular polysaccharide synthesis) that acts as a global regulatory network [107, 108]. The Rcs cascade is activated by several specific regulatory signals, modulating the expression of different genes [107]. These regulatory signals are sensed by the outer membrane lipoprotein RcsF that interacts with an inner membrane protein IgaA (intracellular growth attenuator), which will induce phosphorylation of the components RcsC and RcsD, and the transcriptional regulator RcsB [108]. Phosphorylated RcsB binds to promoter regions and controls expression of several genes, including T6SS. In *Serratia marcescens *, the T6SS is constitutively expressed and able to kill non-aggressive competitors [109, 110]; however, when *S. marcescens* is incubated with an *Acinetobacter* strain encoding an offensive T6SS, the Rcs system of *S. marcescens* senses damage and responds by increasing its T6SS activity, thus finely regulating the T6SS assembly to induce a defensive response [109]. This response is accomplished by the cognate cytoplasmic transcriptional regulator RcsB, which interacts directly with the RcsB-binding motif present in the promoter region of the T6SS gene cluster [109]. In extraintestinal pathogenic *E. coli *, the T6SS activity induced by the antibiotic polymyxin B is controlled by the response regulator RcsB (Fig. 2) [80].

Regulation of genes via QS is typically mediated by two mechanism: (1) a transcriptional regulator, usually from the LuxR-family (luminescence regulator), containing a binding domain for QS molecules and a DNA-binding domain that either induces or represses transcription of target genes upon interaction with the ligand; (2) QS molecules induce a phosphorelay cascade in which an outer membrane sensor interacts with the QS molecule and activates or inhibits a downstream phosphorylation cascade to regulate gene expression (Fig. 2) [111].

*A. hydrophila* and *E. cloacae* control the expression of their T6SS clusters via a QS system in which the quorum signalling molecule N-acylhomoserine lactone (AHL) binds to the transcriptional regulator SdiA (suppressor of division inhibition) – a LuxR homologue – and induces the secretion of T6SS effectors [76, 78]. *C. violaceum* T6SS activation is dependent on a QS system in which the LuxR-type transcriptional regulator CviR binds to homoserine lactone (HSL)-type molecules to promote killing of competitor cells at high cell density [77]. In *B. cenocepacia,* the LuxR homologues CepR and CciR up- and downregulate the T6SS upon binding to AHL, respectively [112, 113]. In *P. aeruginosa,* the regulator MvfR (multiple virulence factor regulator) from the 4-hydroxy-2-alkylquinolines (HAQ) QS system, and the QS HSL-transcription factor LasR (regulator of *lasB*) were shown to repress H1-T6SS but induce H2- and H3-T6SS [79, 114]. QslA (QS LasR-specific anti-activator) is a repressor of the LasR regulator and leads to *P. aeruginosa* H2-T6SS repression [115]. LasR transcription is controlled by the phosphate sensor TCS PhoR/PhoB [116, 117]. Under low phosphate concentrations caused by mutation on the PitA phosphate transporter, PhoB is phosphorylated and promotes expression of QS regulators, which positively regulate the H2- and H3-T6SS of *P. aeruginosa* [65, 116, 117].

In *Vibrio* spp., QS system are key players in the regulation of T6SS expression. The main components are the sensor kinases CqsR (cholerae quorum-sensing receptor), CqsS (cholerae quorum-sensing sensor), VpsS (* Vibrio* polysaccharide biosynthesis sensor) and the LuxPQ complex that phosphorylates the phosphotransferase protein LuxU at low cell density, which then phosphorylates the transcriptional regulator LuxO [118, 119]. LuxO promotes the expression of the regulatory small RNA (sRNA) Qrr1-4 that ultimately inhibits the expression of the transcriptional regulator HapR (TetR-type), which is an activator of the T6SS gene cluster [118, 120–122]. At high cell density, LuxPQ interacts with the autoinducer 2 (AI-2) to inhibit the phosphorylation cascade and activate the T6SS [119, 120]. In *Vibrio fluvialis,* expression of the T6SS2 is controlled by the cholera autoinducer 1 (CAI-1) and AI-2 that interact with the sensors CqsS and LuxP, respectively, preventing LuxU phosphorylation and allowing upregulation of the global transcriptional regulator HapR to induce T6SS expression [123]. *V. fluvialis* T6SS2 upregulation is also mediated by a LuxR homologue, VfqR (* V. fluvialis* quorum-sensing regulator), which promotes gene expression upon binding to AHL [124]. In *V. parahaemolyticus,* the sRNAs Qrr1-4 promote activation of the QS regulator AphA (activator of *tcpP* and *tcpH* expression) to repress the antibacterial T6SS1 at low cell density together with the transcriptional regulator ToxR (toxin regulator) [73]. Conversely, at high cell density, *V. parahaemolyticus* T6SS1 is repressed by the TetR-like QS regulator OpaR (opacity regulator), but T6SS2 is upregulated [48, 73]. In *Vibrio alginolyticus *, an unknown external signal induces the phosphorylation of the cytoplasmic proteins PppA (putative protein phosphatase A) and of VtsR (* Vibrio* type six secretion regulator) via PpkA2 (putative protein kinase A), leading to upregulation of the TetR-type global transcriptional regulator LuxR (different from quorum-sensing LuxR homologues that are LuxR-type) that upregulates the T6SS2 (Fig. 2) [125, 126]. In *V. alginolyticus *, the LysR-type transcriptional regulator VqsA (* Vibrio* quorum-sensing activator) also upregulates the LuxR (TetR-like) global regulator to induce T6SS2 activation [75].

Another factor controlling T6SSs is bacterial growth phase. In species like *V. fluvialis *, *P. aeruginosa* and *P. putida *, the T6SS is upregulated at the stationary phase [64, 89, 127, 128]. The *P. aeruginosa* H2-T6SS is positively regulated during the stationary phase by HSL-dependent signalling [64]. In *V. cholerae *, the growth phase-dependent activation of T6SS occurs at the end of the exponential phase and requires the QS-response regulator HapR [122].

Another important aspect of T6SS activation regulated by QS systems is the control of bacterial community cheaters. Cheaters are cells within a community that do not produce public goods but consume the resources synthesized by neighbour cells [129]. These cheaters are usually mutants of QS receptors and the T6SS act as a mechanism to control this subpopulation. Such control is observed in *B. thailandensis* via the QS receptor BtaR1 (sensing AHL) that regulates the production of biofilm. Bacteria that stop responding to BtaR1 turn into cheaters and stop expressing the T6SS immunity proteins, becoming susceptible to T6SS killing by their siblings [41]. This control is fundamental for the success of the species as proliferation of cheaters can negatively affect the whole population [130]. In *P. aeruginosa,* mutation of the LasR regulator is known to result in the production of cheaters [131]; and in *Vibrio* spp., mutations in components of the QS pathway involving LuxPQ and the global regulator HapR also lead to the production of cheaters [132, 133].

The T6SS activity can also induce QS-related mutations. In *V. cholerae,* it is possible to observe colony sectoring characterized by different opacities. This heterogeneity is caused by the activity of the T6SS that selects bacteria encoding QS-inactivating mutations in the LuxO pathway [134]. These mutations induce the production of Vsp (* Vibrio* polysaccharide), which functions as a defence mechanism against the T6SS, while simultaneously inhibiting the expression of the T6SS [134, 135]. Such selection promotes genetic variations in the populations at natural habitats and during infection [134].

There are several transcriptional regulators belonging to different families of regulators for which the specific mechanism of T6SS activation is unknown. Like most transcriptional regulators, these families encode a conserved helix-turn-helix (HTH) DNA-binding domain [136], with some families encoding an additional ligand-binding domain that interacts with small molecules (e.g. amino acids, cyclic nucleotides or ions) to regulate conformation changes allowing DNA binding. Examples of groups of transcriptional regulators include Lrp-family (leucine-responsive regulatory protein), IclR-family (isocitrate lyase regulator) and LysR-family [137–139]. OscR (osmolarity controlled regulator) from the IclR-family represses the T6SS of *V. cholerae* O1 at low osmolarity [44]. TasR (type VI associated transcriptional regulator) from the Lrp-family induces the T6SS2 of *V. fischeri* in environments with high viscosity [50, 140]. In *V. parahaemolyticus,* TfoY (TfoX/Sxy family) controls expression of T6SS1 under warm marine-like conditions possibly by upregulating the Lrp-family regulator (VP1407) encoded in the T6SS cluster [47, 48], while the CalR (LysR-type) upregulates the T6SS2 [48, 141]. The AraC-type transcriptional regulator AggR from enteroaggregative *E. coli* induces the antibacterial T6SS-3 (Fig. 2) [142–144].

Some T6SSs are controlled by transcriptional factors encoded in non-chromosomal elements such as plasmids. Strains of *Acinetobacter baumannii* carry large conjugative plasmids (LCPs) that are important for spreading antibiotic resistance [145–147]. These plasmids encode TetR-like (tetracycline repressor) transcriptional regulators, which are composed by an N-terminal HTH DNA-binding domain and a C-terminal ligand-binding domain that bind to signalling molecules (e.g. antibiotics, fatty acids and metal ions) to regulate gene expression [148]. TetR-like regulators from *A. baumannii* LCPs suppress expression of its offensive T6SS [145], thus preventing bacterial killing and favouring conjugation among neighbour cells to allow plasmid dissemination [145].

Sigma factors and enhancer-binding proteins (EBP) are another group of transcriptional regulators controlling T6SS expression. Sigma factors are subunits of the RNA polymerase responsible for the recognition of promoter sequences [85]. EBPs are activators of the sigma factor σ^54^ and promote ATP hydrolysis to induce structural alterations of the RNA polymerase holoenzyme bound to DNA to help with transcription [149]. In *V. cholerae *, the first pair of sigma factor and EBP was described, which comprise RpoN (σ^54^) and VasH (virulence-associated secretion protein) that promote transcription of T6SS genes [150, 151]. VasH homologues also control the expression of T6SSs in *A. hydrophila* [152, 153] and *V. fischeri* [154]. Another EBP possibly interacting with RpoN is VP1391 from *V. parahaemolyticus *, which is required for Hcp expression under marine-like conditions [48]. Additionally, the EBP FleQ from *Pseudomonas* spp. is a positive regulator of the F1- and F3-T6SS from *P. fluorescens* [155], but a repressor of the K1-T6SS from *P. putida* [89]. RpoN-dependent expression was also described for the T6SSs of *Plesiomonas shigelloides* [156]*, P. putida* [157]*, K. pneumoniae* [51] and *P. aeruginosa* [158–160]. Moreover, additional sigma factors positively participate in T6SSs expression such as RpoS (σ^38^) in *K. pneumoniae* [51] and *Yersinia pseudotuberculosis* [161] and RpoE (σ^24^) in *Pseudomonas plecoglossicida* promotes T6SS-2 expression [162]. Conversely, RpoS represses K1-T6SS from *P. putida* [89]. It is important to point out that sigma factors are global regulators controlling several genes and phenotypes. RpoN is an inducer of flagellar motility in *V. cholerae* [163], while RpoS also controls motility and response to several abiotic stressors in *Y. pseudotuberculosis* [161].

An important mechanism of T6SS repression in mediated by histone-like nucleoid structuring proteins (H-NS) (Fig. 2). H-NS control the expression of genes acquired by horizontal gene transfer that are rich in AT nucleotides [164]. T6SS expression can be repressed by H-NS in *A. baumannii* [165], *V. parahaemolyticus* [48] and *S*. Typhimurium [166]. In *P. aeruginosa,* the H-NS homologue MvaT represses the H2- and H3-T6SS [167], while the MvaT-paralogue TurA represses the K2-T6SS from *P. putida* [168]. *V. cholerae* O1 encodes a protein with low similarity to H-NS (TsrA, type VI secretion system regulator A), which is also associated with T6SS repression [74].

It is worth mentioning that orphan/auxiliary T6SS gene clusters are often coregulated with the main cluster. An example is the *V. cholerae* auxiliary clusters 1 and 2 that are under the control of RpoN and VasH, which is encoded within the main T6SS cluster, hence the auxiliary clusters are only expressed after the main cluster is induced [163]. Another example is the upregulation of *P. aeruginosa* H2-T6SS and its cognate orphan clusters by RpoN and Sfa2 (sigma factor activator 2). Sfa2 is encoded within the main cluster and controls the expression of both the main H2-T6SS cluster and the orphan genes [159]. Certain orphan effectors are upregulated under specific conditions, such as the effector TseT secreted via the H2-T6SS of *P. aeruginosa *, which is upregulated by high iron concentration during viral co-infections [169]. In addition, effectors and orphan clusters can be self-regulated. An interesting case is the effector CccR from *Y. pseudotuberculosis *, which displays dual function both as toxin and transcriptional factor [170]. CccR presents an N-terminal FIC domain (filamentation induced by cAMP) and a C-terminal HTH domain [170]. In non-self cells, CccR acts as a toxin causing adenylation of the cell division protein FtsZ and inducing cell filamentation and growth arrest. In cells of the same species, CccR binds to the *cccR* promoter and acts as a negative transcriptional regulator [170]. Similarly, the immunity proteins TsiTBgs from *Burkholderia gladioli* encode a HTH DNA-binding domain and act as transcriptional regulators to inhibit the expression of the operon containing their cognate effectors TseTBgs [171].

## Posttranscriptional regulation

The regulation at the posttranscriptional level is achieved via RNA-binding proteins or small regulatory RNAs that bind to mRNAs to control their stability and translation [172, 173]. These regulatory components are controlled by upstream signalling pathways that sense environmental signals, such as two-component and quorum-sensing systems. The most well-studied examples of posttranscriptional regulation of T6SSs are from *P. aeruginosa* and *Vibrio* spp.

*P. aeruginosa* H1-, H2- and H3-T6SSs, *P. putida* K1-T6SS and *P. syringae* T6SS transcripts are all posttranscriptionally regulated via the RNA-binding proteins RsmA and RsmN (repressor of secondary metabolites) [88–90, 174]. These proteins can independently bind to the 5′ untranslated region of T6SS mRNAs with a GGA motif to block the ribosomal-binding site and prevent translation [88, 175, 176]. Binding of RsmA/N to target mRNAs is controlled by the small regulatory RNAs RsmZ and RsmY, which also contain GGA motifs and compete with the targets by sequestering RsmA/N (Fig. 2) [174]. The upstream regulatory pathway of RsmA/N is incredibly complex. RsmY/Z are regulated by many components: (1) the atypical TCS GacS/GacA in which GacS is regulated by the sensor kinases LadS (lost adherence) and RetS (regulator of exopolysaccharides and type III secretion) [177]; (2) the RNase YbeY [178]; (3) the pyocin regulator PrtR, an λCI (phage λ protein CI) homologue [179, 180]; (4) the transcriptional regulator NrtR (Nudix-related transcriptional regulator) [181, 182]; (5) the RNA-binding protein RtcB (RNA terminal phosphate cyclase) [183]; and (6) the transcriptional regulator from the TCS AlgZ/AlgR (alginate biosynthesis) [184]. The histidine kinase GacS is repressed by RetS via the formation of hetero-oligomers that prevent GacS phosphorylation [185]. After an unknown stimulus, LadS phosphorylates and activates GacS [186], which then phosphorylates GacA to promote the expression of RsmY/Z that will induce translation of T6SS mRNAs [175]. YbeY is a RNase associated with ribosomal and small RNA maturation and is involved in the repression of RetS [178]. During the maturation stage of the biofilm, GacS and GacA expression is inhibited by PrtR [180]. NrtR represses RsmY/Z expression to inhibit the translation of T6SS transcripts while also directly repressing T6SS gene cluster promoters [181]. A *rtcB* mutant presented increased levels of RsmA, possibly implicating RtcB in metabolism of RsmY/Z [183]. The AlgR regulator activates the expression of RsmA independently of phosphorylation via its cognate histidine kinase AlgZ (Fig. 2) [184].

The Gac/Rsm system is fundamental for the activation of the *P. aeruginosa* response to antagonism (PARA) in which cells can sense danger signals from lysed kin cells after encounters with T6SS^+^ or T4SS^+^ competitors [187]. Activation of this response is independent of the threonine phosphorylation (TPP) pathway (described below) [187], and relies on the sensing of an unknown cytoplasmic danger signal via Gac/Rsm system, which leads to transcriptional and posttranscriptional activation of H1-T6SS [187]. In addition, most of the components of the Gac/Rsm pathway described above are responsible for modulating gene expression during biofilm formation or host infection. The RetS-GacS-LadS system regulates the expression of genes related to exopolysaccharides (EPS) production [188], and the upregulation of *P. aeruginosa* T6SSs was observed in biofilm rather than planktonic states [114, 189]. Likewise, PrtR is related to pathogenesis and survival to antibiotics in biofilms [190]. The shared regulatory pathways of T6SSs with the formation of biofilms and expression of host virulence factors highlights the importance of their coordinated action for bacterial fitness. An example of this fine coordination is the switch between sessile and motile lifestyle of *P. aeruginosa* [191]. In this bacterium, higher levels of c-di-GMP are linked to biofilm formation and activation of the H1-T6SS via a pathway dependent on the Gac/Rsm system [191, 192]. Conversely, after decrease of c-di-GMP levels, there is downregulation of the T6SS, increase in bacterial mobility and increase in T3SS activity with the secretion of anti-host effectors [191, 193].

In *V. cholerae *, the QS-induced cascade involving LuxUO regulates T6SS expression at the mRNA level. At low cell density, sensor histidine kinases LuxQ, CsqS, CqsR or VpsS phosphorylate the phosphotransfer protein LuxU, which then phosphorylates the transcriptional regulator LuxO [118, 119]. LuxO induces the activation of quorum-sensing regulatory small RNAs Qrr1-4 that repress T6SS mRNAs by direct binding to transcripts, while also inhibiting the master regulator HapR to prevent T6SS transcription [118, 120]. At high cell density, the signalling cascade is not activated and T6SS mRNAs are undisturbed (Fig. 2) [120].

In *V. cholerae* O1*,* it was described that CspV (cold-shock protein) is necessary to maintain high levels of Hcp mRNAs upon cold shock and for antibacterial T6SS activity at 25 and 37 °C; however, no direct interaction between CspV and T6SS mRNA transcripts was demonstrated [45]. Csp proteins are nucleic acid-binding proteins that usually bind to RNAs to ensure correct transcription and translation during cold shock [194]. In addition, CspV regulates other phenotypes such as bacterial attachment to zooplankton [45], and thus could be indirectly mediating the T6SS activation via other regulatory pathways (Fig. 2).

Additionally, an indirect form of posttranscriptional regulation occurs via riboswitches, which are 5′-untranslated regions of mRNAs that control translational upon binding to specific ligands [195]. In *V. cholerae,* the mRNA of the T6SS transcriptional activator TfoY contains the riboswitch Vc2 that is controlled by c-di-GMP and prevents translation by altering the mRNA secondary structure and masking the ribosomal-binding site [196–198]. Thus, low concentrations of c-di-GMP allow the increase of TfoY protein levels, which leads to T6SS expression [196, 199].

## Posttranslational regulation

The last steps in T6SS regulation depend on posttranslational modifications to modulate protein levels and activity. In prokaryotes, several types of modifications participate in regulation of protein function, such as deamination, acetylation, glycosylation, carboxylation, methylation and phosphorylation [200]. Phosphorylation is a key modification for the assembly of T6SS apparatus. After the synthesis/translation of individual T6SS structural components, these must be organized into the subcomplexes: membrane, baseplate and tail (Fig. 1a). This step determines the cellular location in which a T6SS apparatus will be assembled [201].

An important posttranslational regulatory pathway is the threonine phosphorylation (TPP) first described in *P. aeruginosa* [202]. The TPP pathway is conserved among other species, such as *S. marcescens* and *A. tumefaciens* [203–205]. In these bacteria, the membrane associated kinase PpkA initiates the signalling cascade via dimerization and autophosphorylation of its conserved serine residue, followed by phosphorylation of a threonine reside in a cytoplasmic target protein to promote T6SS assembly [201]. Each bacterium has a particular activation mechanism and phosphorylates a specific cytoplasmic target.

For the defensive H1-T6SS of *P. aeruginosa *, the regulation of the TPP pathway begins with the outer membrane protein TagQ (type VI accessory gene Q) and the periplasmic protein TagR (type VI accessory gene R). TagQ in association with TagR phosphorylates the ABC-transporter-like TagTS [206], which leads to PpkA activation and autophosphorylation. Activated PpkA interacts with the cytoplasmic protein Fha1 (Forkhead-associated domain) to promote its phosphorylation [202] and induce T6SS assembly [33, 202, 206, 207]. TagQRST proteins are responsible for sensing membrane damage and guiding the location in which the T6SS apparatus will be assembled [33, 35–37].

In the offensive T6SS of *S. marcescens,* TPP pathway is initiated by the periplasmic protein RtkS (regulator of T6SS kinase in *Serratia *), which activates PpkA and induces the downstream phosphorylation of Fha to induce the assembly of the T6SS [204]. For both *S. marcescens* and *P. aeruginosa,* phosphorylated Fha is required for a functional T6SS [202, 204]. In these two species, subsequent dephosphorylation of Fha is carried out by the phosphatase PppA, which inhibits T6SS assembly [33, 202]. The presence of PppA was shown to be fundamental for the re-orientation and distribution of T6SSs along the cell perimeter to ensure efficient bacterial killing [33, 204]. The PpkA, PppA, Fha and TagQRTS proteins are also encoded in the antibacterial F1-T6SS cluster of *P. fluorescens* and could function similarly to the *P. aeruginosa* system [155].

Other bacterial species contain posttranslationally regulated T6SSs but do not seem to encode the full PpkA-Fha-PppA system. In these cases, PpkA phosphorylates additional T6SS structural components besides FHA-containing proteins. In *A. tumefaciens,* an unknown protein promotes PpkA-mediated phosphorylation of the T6SS membrane protein TssL. This modification is fundamental for the formation of the TssM-TssL complex to allow its interaction with Hcp [205]. Phosphorylated TssL also interacts with Fha2 of *A. tumefaciens* and this association is necessary for T6SS activity [205]. In *V. alginolyticus,* PpkA2 phosphorylates TssL, which leads to Fha recruitment to the membrane complex for the activation of T6SS2 [126]. *V. cholerae* does not encode PppA or PpkA proteins but the FHA domain-containing protein TagH (type VI accessory gene H) is necessary for T6SS activity as its mutant does not secrete Hcp; however, phosphorylation of the FHA domain of TagH was not observed and its interaction with other T6SS structural components was not determined [208]. It is possible that TagH functions by interacting with other phosphorylated T6SS components, which are modified by unidentified kinases (Fig. 2) [208].

In addition to the phosphorylation pathways, *P. aeruginosa, S. marcescens* and *A. tumefaciens* encode the accessory protein TagF, which negatively regulates T6SS activity by preventing its assembly [204, 205, 209, 210]. In *P. aeruginosa *, TagF sequesters the Fha1 protein to prevent downstream activation of the T6SS [210]. In *S. marcescens,* TagF prevents the formation of the T6SS membrane complex, possibly by direct interacting with the structural component TssM, which is often encoded upstream of *tagF* in the operon [204]. *A. tumefaciens* encodes the chimeric protein TagF-PppA in which the TagF portion inhibits T6SS activity by binding to Fha, while the function of the PppA portion is uncertain as it does not dephosphorylate PpkA targets (Fig. 2) [210].

The assembly of the T6SS machinery is also spatially and temporally regulated by contact between cells. In *Acinetobacter baylyi, A. baumannii*, and *B. thailandensis,* a portion of their constitutive T6SSs apparatus assemble at the same site where there is contact between cells [211]. The outer membrane protein OmpA was proposed to sense the contact between cells and signals via the periplasmic protein TslA (type 6 secretion dynamic localization protein A), which initiates the assembly of the membrane complex via TslA-TssM interaction (Fig. 2) [211].

Another interesting aspect on the regulation of T6SS assembly is the control of the sheath contraction. In *E. coli* and *V. cholerae* V52*,* TssA initiates polymerization of the tail components via interaction with the sheath (TssC) and inner tube (Hcp) components at the distal end of the tail (Fig. 1a) [16]. In this context, TagA functions by anchoring the system to the opposite side of the membrane to control firing [212, 213]. The *tagA* mutant displays early contraction of the sheath (60 % of the cells) compared to the wild-type strain (5 % of the cells), indicating that TagA prevents immediate contraction and maintains the tension of the extended state required for effective T6SS firing [212, 214]. In *P. putida,* the K1-T6SS stabilization is mediated at the baseplate by the accessory protein TagB, while the H1-T6SS of *P. aeruginosa* is stabilized by TagJ [214]. These proteins are important for sheath polymerization in T6SSs with a short-time contraction that do not encode the TagA protein [214]. The TssA proteins from *P. putida* and *P. aeruginosa* are shorter than the TssA from *E. coli* [214]. Longer TssA proteins are associated with TagA and present in T6SSs that remain at the extended conformation for longer periods (long-time residence), such as in *E. coli* and *V. cholerae* [214, 215]. Meanwhile, shorter TssA isoforms associate with TagB or TagJ and belong to systems with immediate contraction after sheath extension (short-time residence), such as the H1-T6SS from *P. aeruginosa* (Fig. 1A) [214, 215].

Another special mode of regulation of T6SS activity could be the control of ClpV activity. The ATPase activity of the flagellar exporter protein FliI of *P. fluorescens* and the T3SS exporter HrcN of *P. syringae* are inhibited upon binding to c-di-GMP [216]. Parallelly, c-di-GMP also interacts with the ClpV homologue, ClpB, of *P. fluorescens* in physiologically relevant conditions, possibly linking c-di-GMP with the repression of T6SS disassembly [216].

T6SS can also be controlled by the intracellular concentration of specific structural components. In *V. cholerae* and *E. coli,* the rapid turnover of the baseplate protein TssE was proposed to be a mechanism to control T6SS activity under nutrient poor conditions [127, 217]. In addition, in *V. cholerae,* high levels of Hcp protein directly interact with the VasH transcriptional regulator to repress the expression of T6SS clusters [218].

## Concluding remarks

The diversity of mechanisms regulating the T6SSs is reflected by the great variety of niches bacteria reside. The temporal and spatial regulation of T6SSs allow bacteria to control the expression and synthesis of the T6SS structural components, its assembly and firing at the most strategic time and location inside the cell to promote either an attack or a counterattack. The assembly of T6SS apparatus was predicted to be a costly investment for bacterial cells, hence its expression needed to be well controlled. However, recent findings indicate no impact on bacterial fitness [219, 220], suggesting that the timely regulation of T6SSs might serve mainly ecological purposes rather than energy costs.

There are still many open questions about the environmental signals and regulatory cascades that control the expression of T6SSs in different species. Although much work has been done on characterizing several transcriptional regulatory mechanisms across species, there is proportionally less information about the regulation of T6SSs at the posttranscriptional and posttranslational levels. Most of the antibacterial T6SSs described to date cannot be classified into offensive or defensive systems due to the lack of knowledge about the conditions that promote their expression. Many T6SSs are regulated via inputs from multiple regulatory networks, which impose a challenge for straightforward experimental assays to identify the conditions that promote full system activation. The continuous work of different groups should shed light on the remaining knowledge gaps about the environmental signals and regulatory elements controlling the expression of T6SSs across several species to help obtain a full picture about the regulation of these systems during interbacterial encounters.
